# Dry-Lensectomy Assisted Lensectomy in the Management for End-Stage Familial Exudative Vitreoretinopathy Complicated With Anterior Segment Abnormalities

**DOI:** 10.3389/fmed.2022.850129

**Published:** 2022-04-29

**Authors:** Jie Peng, Ziwei Zhao, Yihua Zou, Xuerui Zhang, Yuan Yang, Qiujing Huang, Mingpeng Xu, Yu Xu, Peiquan Zhao

**Affiliations:** Department of Ophthalmology, Xin Hua Hospital Affiliated to Shanghai Jiao Tong University School of Medicine, Shanghai, China

**Keywords:** lensectomy, vitrectomy, familial exudative vitreoretinopathy, retinal detachment, secondary glaucoma, capsule-endothelial adhesion

## Abstract

**Purpose:**

To report a modified technique of dry-lensectomy assisted lensectomy in the management of end-stage familial exudative vitreoretinopathy (FEVR) complicated with capsule-endothelial, iris-endothelial adhesion and secondary glaucoma.

**Methods:**

24 eyes of 16 patients with severe complications of advanced pediatric total retinal detachment caused by FEVR who received limbus-based dry-lensectomy were studied retrospectively. Preoperative and postoperative clinical information was collected and reviewed.

**Results:**

Among the 24 eyes, three eyes (12.50%) underwent lensectomy combined with vitrectomy and membrane peeling simultaneously. 21 (87.50%) eyes underwent lensectomy without membrane peeling due to severe corneal opacity or retinal vascular activity, of which eight underwent another vitrectomy combined with membrane peeling. At the last visit (mean:13.86 ± 5.24 months of follow-up), all eyes had a reconstructed anterior chamber with normal depth. Among 21 eyes having preoperative corneal opacity, 15 (71.43%) had a clearer cornea with reduced opacity, 5 (23.81%) showed similar corneal opacification without deterioration. Among 11 eyes undergone retrolental fibroplasia peeling, seven (63.64%) eyes showed partial retinal reattachment in open-funnel type.

**Conclusion:**

Dry-lensectomy offered a simple way to lower the intraocular pressure and simplified the surgery, which helped to solve the severe anterior segment complications and offer a chance for following retrolental fibroplasia peeling and potential visual gain for selected end-stage FEVR patients.

## Introduction

Retinal vascular anomalies are one of the main causes of visual impairment (1). Among them, familial exudative vitreoretinopathy (FEVR) is a major cause of pediatric and juvenile RD in Asian populations (2, 3). FEVR is classified into five stages (4). Complications of advanced FEVR include corneal edema, cataract, secondary glaucoma and so on. Though treated, part of FEVR eyes would suffer from disease progression to retinal detachment (RD). Treatment of stage 5 FEVR is still a tough task with poor anatomical and functional results. Those eyes had no chance for any visual function or even a good appearance if left untreated. Lensectomy alone or combined with vitrectomy helped to save the opacity of cornea secondary to capsule-endothelial adhesion (3) and glaucoma (5). Vitrectomy with membrane peeling for Stage 5 FEVR is beneficial in preventing total blindness in some patients (3). Visual function of light perception is much better than no light perception at all, at least regarding maintaining the circadian rhythm (6).

In stage 5 FEVR eyes with capsule-endothelial or iris-endothelial adhesion, it was hard to separate the iris or/and the lens from the corneal endothelium with viscoelastics. The first difficulty to conquer was the capsule-endothelial adhesion separation and to reconstruct the anterior chamber (AC). Herein, we introduce a modified method to deal with this tough situation.

## Methods

This is a single-center, retrospective, consecutive, and interventional case series. This study was approved by the Ethics Committee of Xinhua Hospital affiliated to Shanghai Jiao Tong University School of Medicine. All procedures were performed in accordance with the Helsinki Declaration.

**Figure 1:**
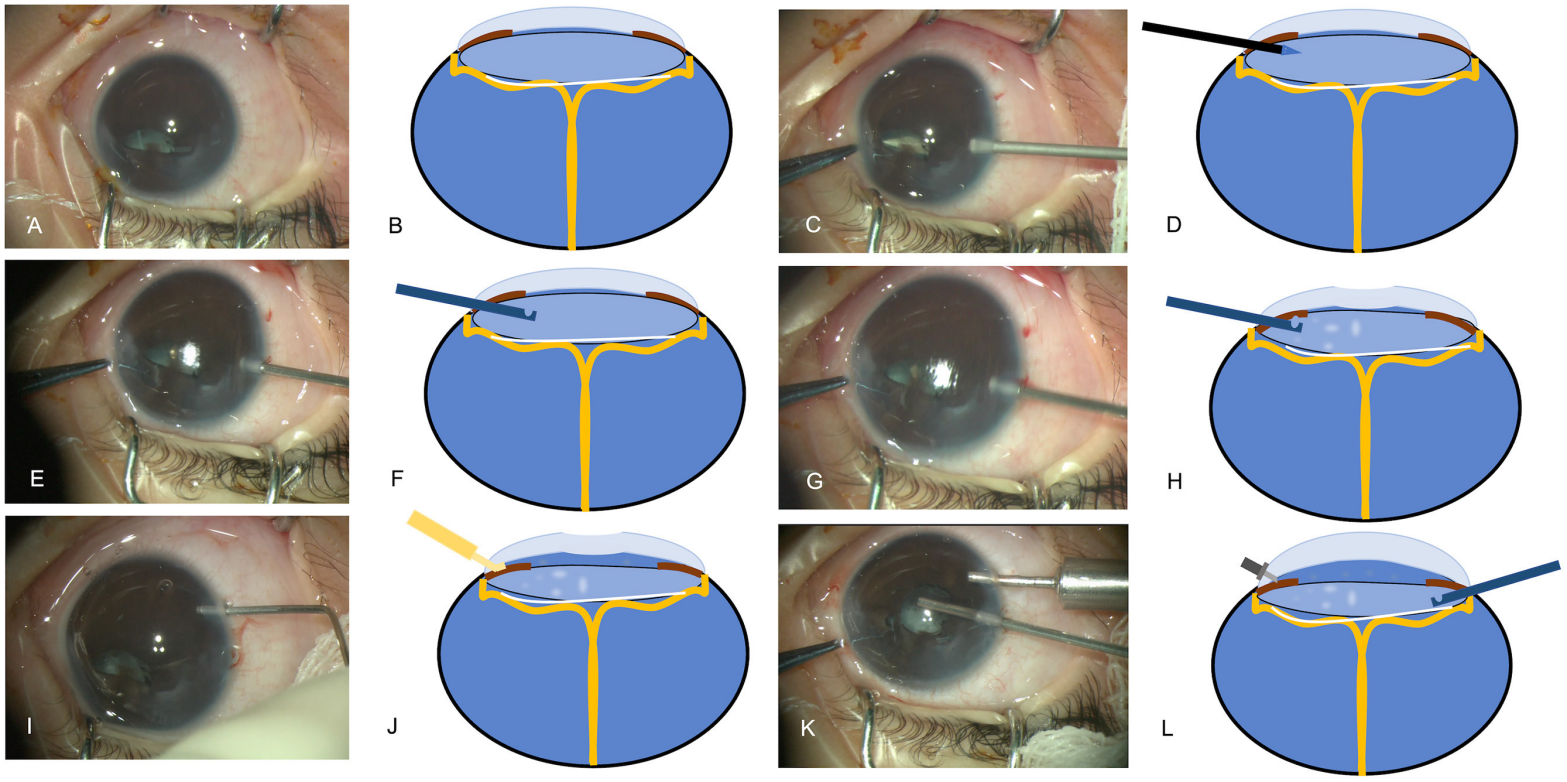
The surgical procedures of dry-lensectomy. **(A,C,E,G,I,K)** Images acquired during the surgery, **(B,D,F,H,J,L)** schematic diagrams of the procedures. **(A,B)** A combination of anterior segment anomalies (anterior lens displacement, marked flat anterior chamber (AC), iridocapsular adhesions, capsule-endothelial adhesion with corneal opacification) was noted; **(C,D)** a limbal incision was made by 20-Gauge MVR blade in a slightly downward manner, starting from the limbus, going through the mid-periphery of the iris and then into the middle of dislocated lens; **(E,F)** the 23-Gauge vitrector went through the same approach into the lens without anterior chamber irrigation and with cutter off; **(G,H)** as the vitrector is in the lens, the vitrector started to do the cutting with suction; **(I,J)** the viscoelastic can be easily injected into the AC to reconstruct the AC; **(K,L)** the AC maintainer was inserted followed by the lensectomy in a traditional way.

Twenty-four eyes from 16 FEVR infants with anterior segment compromise, shallow/flat AC, capsule-endothelial or iris-endothelial adhesion underwent dry-lensectomy-based surgery from April 2020 to September 2021 were studied. The clinical information has been listed in Table 1. The patients underwent comprehensive preoperative and postoperative ophthalmic examinations. The anterior and posterior segments were examined with binocular indirect ophthalmoscopy with a + 20D lens. Wide-field fundus images of the anterior and posterior segments were taken with RetCam III (Clarity Medical Systems, Pleasanton, California, USA). Ultrasound examinations were performed to confirm RDs. Ultrasound Biomicroscope (UBM) was done to assess the AC depth and AC angle, especially for the eyes with severe corneal opacity/edema. Intraocular pressure (IOP) was measured using a rebound tonometer (i-Care, Espoo Finnish). Sedation with oral intake of chloral hydrate (0.5 ml/kg) was provided when needed. In our hospital, only for eyes with total retinal detachment and severe anterior segment abnormalities, such as secondary glaucoma, shallow/flat AC or capsule-endothelial or iris-endothelial adhesion, with/ without corneal macula or keratoleukoma (<2/3 corneal area), and without phthisis bulbi, the surgery was done (Figures 1A,B). The anatomic outcomes and the complications were recorded and analyzed.

**Table 1:** Clinical information of the patients.

**No**.	**Sex**	**Age at surgery**	**GA (weeks)**	**BW (g)**	**Family history**	**Genetic test**	**Stages of FEVR disease**		**Eyes underwent surgery**	**Preoperative IOP (mmHg)**		**Postoperative IOP (mmHg)**		**Preoperative Corneal edema/ opacity**		**Follow-up durations (months)**	**Postoperative Corneal edema/ opacity**		**Eye(s) underwent vitrectomy and RLF peeling**	**Ocular examinations at the last visit**
							**OD**	**OS**		**OD**	**OS**	**OD**	**OS**	**OD**	**OS**		**OD**	**OS**		
1	M	2.1	37	1500	(+)	NDP c.339 insG/chrX-43809107	5	5	OU lensectomy	8	6	5	6	(+)	(+)	21	remained	remained	/	OU pupillary block with reconstructed AC
2	M	2.3	38	2700	(+)	FZD4 C226 G>T/chr11-86665902	5	5	OU lensectomy	10	26	3	7	(–)	(+)	20	(–)	deterio- rated	OU	OD partial retinal reattachment with closed-funnel type; OS pthisis bulbi
3	F	4.4	40	2950	(–)	LRP5 chr11-68177424/c.G2134A	1	5	OS lensectomy	4	6	5	5	/	(+)	20	/	better	OS	OS partial retinal reattachment with open-funnel type
4	M	3.6	40	3300	(–)	NDP c.134_135delTG/chrX-43817757 43817758	4	5	OS lensectomy	6	8	7	9	/	(+)	19	/	better	/	OS pupillary block with reconstructed AC
5	F	3.1	39	3350	(–)	FZD4 chr11-86663485/c.A313G	5	1	OD lensectomy	8	6	7	6	(+)	(–)	18.5	better	/	/	OD pupillary block with reconstructed AC
6	F	3.5	39.6	2500	(–)	FZD4 c.1176_1178 delGGC/chr11 -86662620 86662622	5	5	OU lensectomy	10	11	6	6	(+)	(–)	17.5	better	/	OS	OS partial retinal reattachment with open-funnel type
7	F	4.1	38	3100	(+)	FZD4 chr11 86663050 86663051/c747dupC	3	5	OS lensectomy	7	18	8	10	/	(+)	16.5	/	better	/	OS pupillary block with reconstructed AC
8	M	5.4	40	3750	(+)	ZNF408 chr11-46726357 46726357/c.1083delG	5	1	OD lensectomy	7	6	7	6	(+)	/	15	remained	/	/	OD pupillary block with reconstructed AC
9	M	2.1	37	2230	(–)	NDP chrX-43817758/c.134T>G	5	5	OU lensectomy	9	8	8	5	(+)	(+)	12.5	remained	remained	/	OU pupillary block with reconstructed AC
10	M	2	38	4030	(–)	NDP chrX-43809104/c.343C>T	5	5	OU lensectomy	4	5	5	5	(+)	(+)	11.5	better	better	OU	OD partial retinal reattachment with open-funnel type; OS partial retinal reattachment with closed-funnel type;
11	F	2.2	39	2840	(–)	LRP5 chr11-68174159/c. 1969A>G	5	5	OU lensectomy	7	5	5	8	(+)	(+)	11	better	better	OD	OD partial retinal reattachment with open-funnel type;
12	F	3.8	37	3100	(–)	LRP5 chr11-68115513/c. C290T	5	5	OU lensectomy +vitrectomy +RLF peeling	3	4	6	4	(+)	(+)	10.5	better	better	OU	OU partial retinal reattachment with open-funnel type with posterior pole nearly reattached.
13	M	5.8	38	2600	(+)	TSPAN12 chr7-120478883/c. 233G>A	4	5	OS lensectomy	7	6	8	4	/	(+)	9	/	better	/	OS pupillary block with reconstructed AC
14	M	1.9	39	3250	(–)	FZD4 chr11-86662209/c. G1589A	1	5	OS lensectomy	6	7	6	5	/	(+)	8	/	better	OS	OS pupillary block with reconstructed AC
15	M	4.6	41	4600	(–)	LRP5 chr11-68115489/c. 266A>G	5	4	OD lensectomy	9	9	8	10	(–)	/	7.5	/	/	/	OD pupillary block with reconstructed AC
16	M	3.3	42	3850	(–)	NDP chrX-43809162/c. 285C>G	5	5	OD lensectomy. OS lensectomy + vitrectomy + RLF peeling	6	7	4	9	(+)	(+)	4.3	better	better	OS	OS partial retinal reattachment with open-funnel type with posterior pole nearly reattached.

*No, number; M, male; F, female; GA, gestational age; BW, birth weight; g, gram; OD, oculus dexter; OS, oculus sinister; OU, oculus uterque; FEVR, familial exudative vitreoretinopathy; AC, anterior chamber; RLF, retrolental fibroplasia*.

### Statistical Analysis

Statistical analysis was performed using Statistical Package for the Social Sciences (SPSS) Version 20 (IBM Corp, Armonk, NY, USA). Data were expressed as the mean ± standard deviation or as median and range. A paired-*t*-test was used to compare preoperative and postoperative IOP. Regression analysis was applied to analysis the associations between the factors (sex, gestational age, birth weight, age at the surgery, family history, preoperative IOP, postoperative IOP, existence of preoperative corneal oedema/ opacity) and the postoperative corneal status (0 = deterioration of corneal opacity; 1 = remained the same corneal opacity; 2 = reduced corneal opacity;3 = transparent cornea). A *P*-value was considered to be statistically significant if *P* < 0.05.

### Surgical Techniques

Dry-lensectomy assisted lensectomy and closed-vitrectomy were performed via limbus approaches. A limbal incision was made by 20-Gauge MVR blade in a slightly downward manner. The route of the blade started from the limbus, went through the mid-periphery of the iris and then into the middle of dislocated lens, which can be easily observed under the microscope (Figures 1C,D). After this incision, 23-Gauge vitrector went through the same approach into the lens without AC irrigation and with cutter off (Figures 1E,F). As the vitrector is in the lens, the vitrector started to do the cutting with suction (cutting rate: 4,000 bpm, Vacuum: 500 mmHg), and this maneuver was called dry-lensectomy. After removal of some part of the crystalline lens, which helped to reduce the volume of intraocular content, the eye was soft with lower IOP. At this moment, Descemet's membrane folds or a flat or umbilicate cornea could be observed (Figures 1G,H). At this time, the viscoelastic can be easily injected into the AC, separating the capsule-endothelial, iris-endothelial, and capsule-iris adhesion, and reconstructing the AC (Figures 1I,J). At that, the AC maintainer could be inserted followed by the lensectomy in a traditional way (Figures 1K,L). The opaque corneal epithelium can be removed to offer better visualization. Vitrectomy and membrane peeling were performed only when the central part of cornea is clear and lack of retinal vascular activity. Otherwise, a second vitrectomy with membrane peeling was performed on a quiet eye several months later as our previous study (3). Iris speculum was used for retrolental fibroplasia (RLF) dissection as our previous study (7). Intraocular triamcinolone acetonide injection was applied to prevent postoperative inflammatory reactions.

## Results

Eight patients had bilateral surgeries and eight had unilateral surgery. The mean age at the surgery was 3.39 ± 1.24 months old, the mean gestational age was 38.91 ± 1.47 weeks, the mean birth weight was 3103.13 ± 748.41 grams, and the mean follow-up duration was 13.86 ± 5.24 months. Five (31.25%) patients had positive family history. All patients had genetic analysis and the FEVR diagnosis was confirmed.

Dry-lensectomy followed by AC reconstruction was successfully applied in all eyes without iatrogenic retinal holes, Descemet's membrane detachment or conversions to other surgical methods, such as external drainage of subretinal fluid to reduce the IOP.

Among the 24 eyes, three eyes (3/24, 12.50%) underwent lensectomy combined with vitrectomy and membrane peeling at the same surgery. Twenty-one (21/24, 87.50%) eyes underwent lensectomy due to the severe opacity of the cornea or vascular activity of the detached retina. All 21 eyes showed pupil block after the initial lensectomy. Among the 21 eyes, 8(38.10%) eyes had another vitrectomy and membrane peeling at the second time.

**Figure 2:**
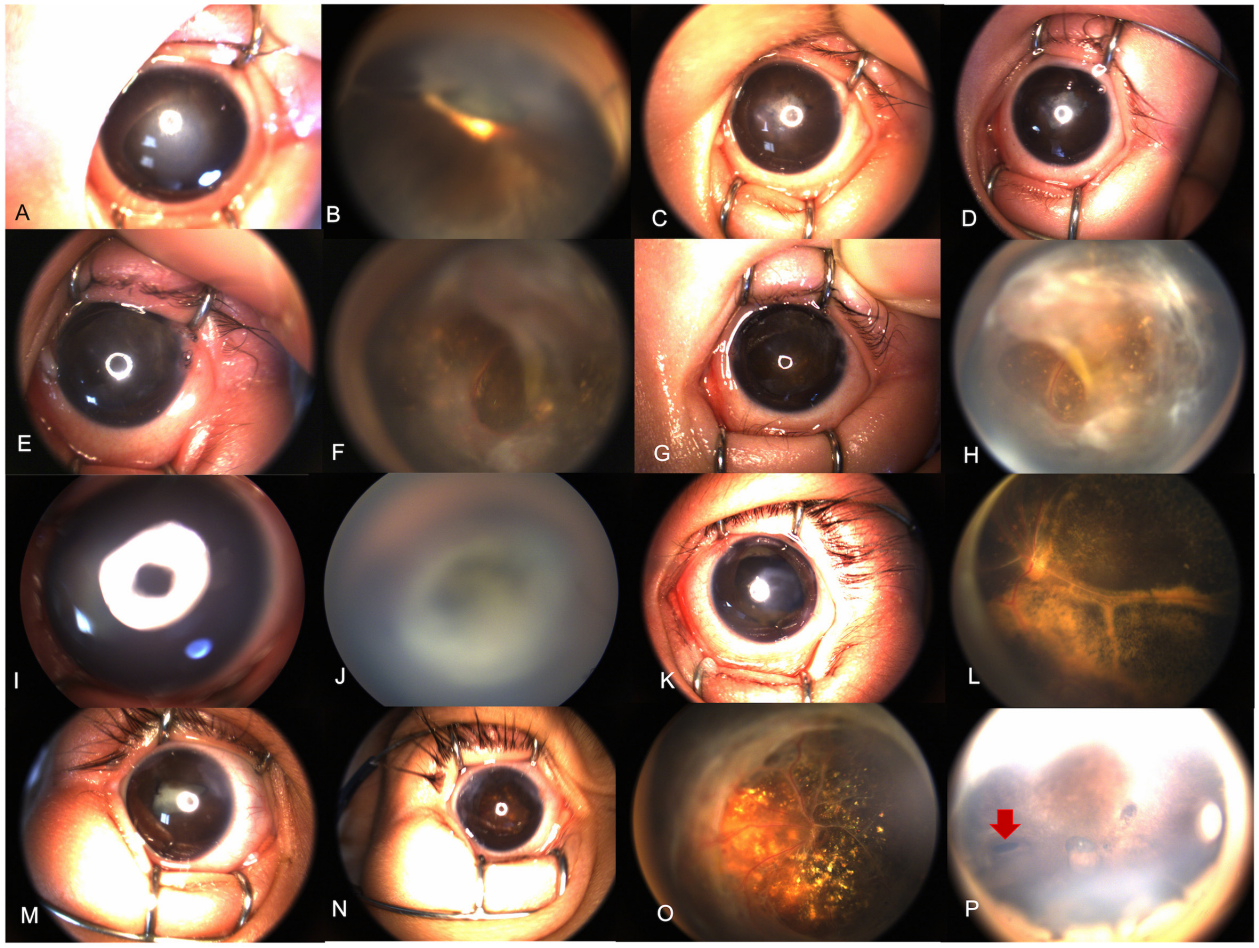
Results of the treatment. **(A**–**H)** Retcam images of a 4-month-old FEVR patient (Case 3); **(A)** a combination of anterior segment anomalies (anterior lens displacement, marked AC shallowing, iridocapsular adhesions, capsule-endothelial adhesion with central corneal opacification) were noted; **(B)** 1-month after the dry-lensectomy assisted surgery, the depth of AC was normal with pupil occlusion; **(C)** 3 months after the dry-lensectomy assisted surgery, the corneal opacity was reduced; **(D)** 8 months after the dry-lensectomy assisted surgery, the corneal is nearly transparent, and a second vitrectomy and RLF peeling was performed. **(E,F)** 1 week after RLF peeling, the retina was partially reattached in an open-funnel manner; **(G,H)** 6 months after the RLF peeling, the anterior segment was in a stable and good status with clear cornea and round pupil and open-funnel retina. **(I**–**L)** Case 16, a 3-month-old male presented with bilateral total RD complicated with corneal opacity and flat AC. Dry-lensectomy combined with vitrectomy and RLF peeling was performed simultaneously. **(K,L)** 4 months after the surgery, the corneal opacity was limited and reduced, with nearly reattached posterior pole; **(M**–**O)** case 12, a 3-month-old FEVR girl received dry-lensectomy assisted surgery and RLF peeling; **(M)** before the surgery; **(N,O)** 7 months postoperatively, the corneal opacity was limited and reduced with round pupil and nearly reattached posterior pole. Subretinal exudates were noted; **(P)** case 11, 7 months after dry-lensectomy assisted lensectomy, the peripheral iridectomy could be seen (red arrow), helping to prevent secondary angle closure or glaucoma caused by pupil block.

**Figure 3:**
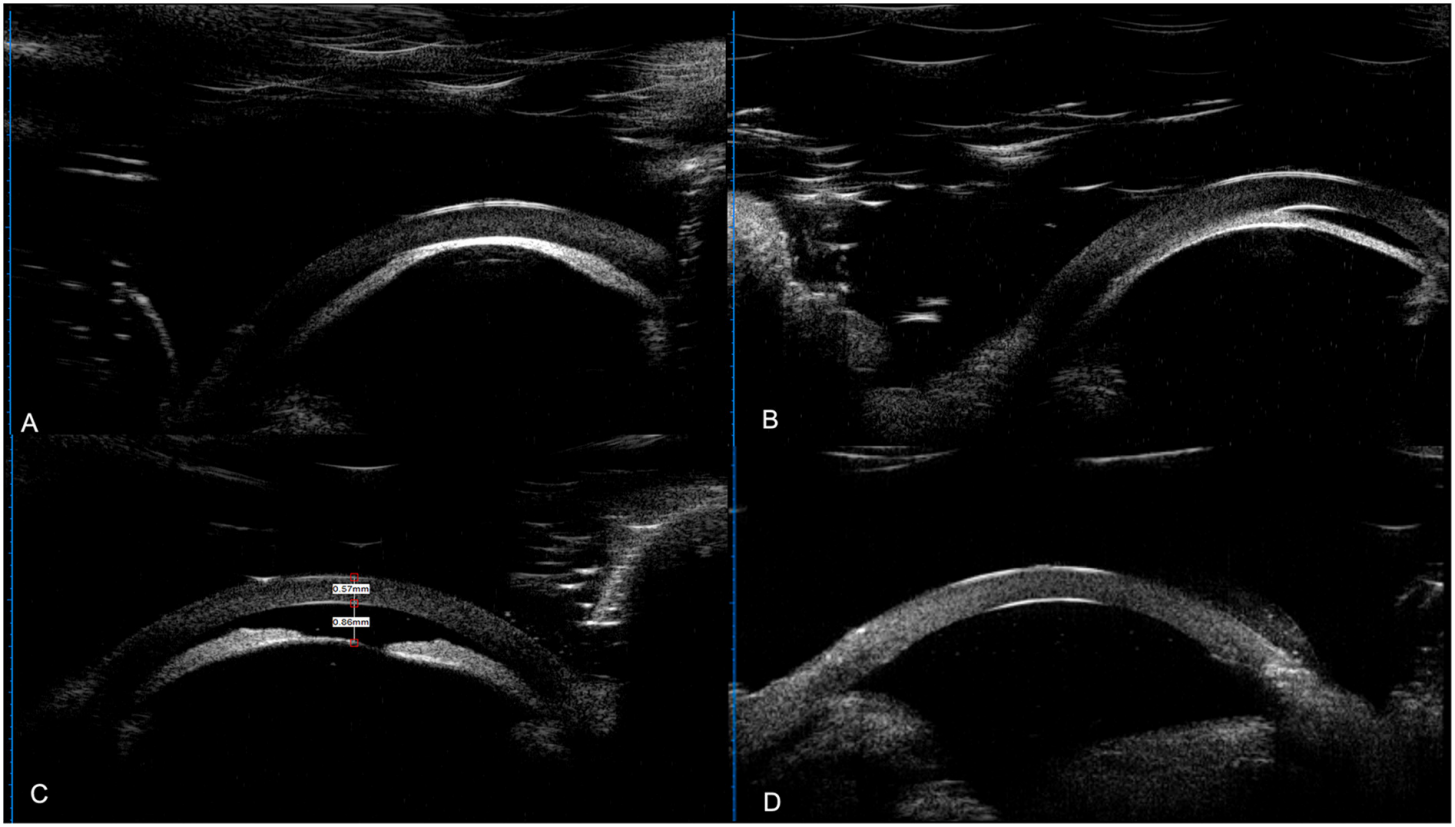
UBM results of the patients. **(A)** UBM result of left eye of case 3 (a 3-month-old female's); **(B)** UBM result of right eye of case 8 (a 5-month-old male). A&B: anterior lens displacement, iridocapsular adhesions, capsule-endothelial adhesion, corneal thickening and edema, and obliterated anterior chamber. **(C,D)** UBM result the right eye of case 15 (a 4-month-old male). **(C)** Preoperative UBM images showed shallowing of the AC with a depth of 0.86 mm, and anterior lens displacement. **(D)** 7 months postoperatively, the AC was reconstructed with a depth of 3.2 mm with AC angle open in this aphakic eye.

The mean preoperative IOP was 8.25 ± 4.83 mmHg. The mean postoperative IOP was 6.12 ± 1.83 mmHg, which is slightly lower than preoperative ones with statistical significance (*P* = 0.03). At the last visit (mean: 13.86 ± 5.24 months of follow-up), all eyes had a reconstructed AC. Twenty-one eyes (21/24, 87.50%) had preoperative corneal edema/opacity, among which 15 (15/21, 71.43%) eyes had a clearer cornea with reduced opacity, five (5/21, 23.81%) showed similar corneal opacification without deterioration and one (1/21, 4.76%) showed progression of corneal degeneration due to pthisis bulbi even with reconstructed AC. The surgical results were shown as Figure 2. UBM examinations were performed on 4 eyes of 3 patients (Figure 3).

In total, 11 eyes had undergone membrane peeling of the RLF, seven (7/11, 63.64%) eyes showed partial retinal reattachment with open-funnel type (three eyes showed nearly reattached posterior pole), two (2/11, 18.18%) showed partial retinal reattachment with closed-funnel type, one (1/11, 9.09%) showed pupil block and one (1/11, 9.09%) showed pthisis bulbi. Visual acuity test was not performed due to patients' incompatibility.

Regression analysis showed female (*t* = 3.793, *p* = 0.001), lower preoperative IOP (*t* = −3.395, *p* = 0.003), and larger birth weight (*t* = 2.095, *p* = 0.049) were associated with better postoperative corneal status.

## Discussions

Angle closure glaucoma (ACG) or angle closure is a rare clinical condition in children, which is always secondary condition, such in advanced stage 5 FEVR eyes at the cicatricial stage, which is hard to handle. Retinopathy of prematurity has many overlaps with FEVR, especially in the advanced stages. In 2021, the ICROP defined stage 5C as conditions that total RD with retrolental fibrovascular tissue or closed-funnel detachment and accompanied by anterior segment abnormalities (e.g., anterior lens displacement, marked AC shallowing, iridocapsular adhesions, capsule-endothelial adhesion with central corneal opacification, or a combination thereof) (8), bringing the severe anterior segment complications to our focus again.

ACG is the most frequent complication of RLF, causing pain and opacity of the cornea. The possible causes included anterior displacement of the lens-iris diaphragm due to RLF contraction, pupillary block, and ciliary block (9). Though surgical and functional outcomes of stage 5 FEVR were poor (3, 4). Lensectomy do have its merits, which was needed to cure the glaucoma and the cataract, and to set the eyes in a quiet, pain-free status (10, 11), preventing keratoleukoma and bulbi phthisis (3, 12, 13). In stage 5 FEVR with total RD, the RLF and total RD adhered to the lens, making lensectomy through par plana impossible with high risks of iatrogenic retinal breaks, a traditional limbal approach lensectomy was always performed.

In these eyes with obliterated AC, the lens-iris diagram was extremely anterior-dislocated with lens adhering to the corneal endothelium with angle closure, it was hard to separate the lens and endothelium with viscoelastics with high IOP and impossible to reconstruct the AC and insert the surgical instruments. The first step was to lower the IOP and reconstruct the AC. We tried external drainage of subretinal fluid in several cases first, but this technique had high risks of ora serrata dialysis and iatrogenic retinal breaks. As a similar concept to lower the IOP with volume depression, we came up with this idea with dry-lensectomy without irrigation initially to soften the eye to conquer the problem. After that, the irrigation tube or AC maintainer was inserted through the limbus incision, the whole traditional lensectomy with/without vitrectomy was performed. In this study, all eyes underwent dry-lensectomy as first step and successful surgery was done without intraoperative complications associated with dry-lensectomy, which is an easy and safe method. Besides, in cases receiving merely lensectomy, pupillary block was seen in all cases due to the RLF. The peripheral iridectomy during dry-lensectomy (Figure 2P, red arrow) helped to prevent another secondary angle closure caused by pupillary block.

Staged lensectomy and vitrectomy was recommended alternative in eyes with severe corneal opacity and vascularly activity (3). In our study, the mean age at the surgery was 3.39 ± 1.24 months, which is at the very early stage of the life. At the surgery day, most of the vitreoretinopathy (85.71%, 18/21) were vascularly active in which staged surgeries were scheduled. The AC was reconstructed in all eyes. Despite one eye (4.76%) showed phthisis bulbi after lensectomy and following membrane peeling, the anterior segment was preserved in a relatively stable status in 20(95.23%) eyes. And 71.43% eyes had a reduced corneal opacity, resulting in a better appearance.

The postoperative IOP was slightly lower than preoperative one (*p* = 0.03), though in a normal range or lower due to total RD and soft eyeball of the children. No eye had persistent elevated IOP or ocular pain in the last visit. Lensectomy, starting with dry-lensectomy, helps to reduce the corneal opacity, preserve a better outlook of the eye and offer a good basis for secondary vitrectomy in selected cases.

Besides, in this study, 63.64% of eyes that had undergone membrane peeling of the RLF showed partial retinal reattachment. And 27.27% (3/11) eyes showed nearly reattached posterior pole, which may offer visual gain in these patients. However, vitrectomy combined with RLF peeling was complicated surgery requiring enormous experience. In our opinion, lensectomy, starting with dry-lensectomy, is really recommended as the first step for stage 5 FEVR with anterior segment anomalies, offering potential chance for surgical for RLF peeling by experienced pediatric vitreoretinal surgeons and chances for potential visual gain. Besides, the surgery can be applied in similar clinical situations, such in stage 5C retinopathy of prematurity.

In this small study, female, lower preoperative IOP, and larger birth weight were associated with better postoperative anatomical outcomes. Male patients had more susceptibility for *NDP* mutation, which tended to cause severe diseases (14). Lower preoperative IOP means early surgical intervention without progression to glaucoma with elevated IOP and worse preoperative corneal compromise. However, bias do exist due to small sample size. In our opinion, these patients all presented with advanced end-stage diseases, and early presentation with early treatment should be associated with better outcomes.

In our hospital, we perform surgery with corneal keratoleukoma <2/3 corneal area, and evaluate the AC in this transparent 1/3 corneal area. If the AC was shallow or flat, we will perform the surgery. We do believe this may differ in different hospitals without a universe consensus. This is our one-site experience. A multicenter study with large scale and long-term follow-up is needed.

Limitations include those biases intrinsic to a retrospective study. Preoperative and postoperative UBM results were not acquired for all patients. The follow-up duration was relatively short and lack of assessments of visual outcomes. Besides, the study sample was small. A further study with longer follow-up, comprehensive ophthalmic examinations and larger scale is needed.

In conclusion, lensectomy, starting with dry-lensectomy, offered a simple way to lower the IOP and made the surgery easier. Lensectomy alone helped to save the opacity of the cornea and offered a chance for following RLF peeling and potential visual gain. It is recommended in the management for eyes with end-stage FEVR or similar situations complicated with marked anterior segment complications.

## Data Availability Statement

The original contributions presented in the study are included in the article/Supplementary Material, further inquiries can be directed to the corresponding authors.

## Ethics Statement

The studies involving human participants were reviewed and approved by Ethics Committee of Xinhua Hospital affiliated with Shanghai Jiao Tong University School of Medicine. Written informed consent to participate in this study was provided by the participants' legal guardian/next of kin.

## Author Contributions

JP, ZZ, YZ, XZ, YY, QH, and MX collected, analyzed, and interpreted the data. JP drafted the manuscript. ZZ, YZ, and XZ carefully revised the manuscript. YX and PZ contributed to the conception of the study. PZ came up with the surgical modification and performed all the surgeries. All authors read and approved the final manuscript.

## Funding

This study was partially supported by Shanghai Sailing Program (Nos. 20YF1429700 and 19YF1432500), the Clinical Research Plan of SHDC (No. SHDC2020CR5014-002) and the National Natural Science Foundation of China (No. 81900908).

## Conflict of Interest

The authors declare that the research was conducted in the absence of any commercial or financial relationships that could be construed as a potential conflict of interest.

## Publisher's Note

All claims expressed in this article are solely those of the authors and do not necessarily represent those of their affiliated organizations, or those of the publisher, the editors and the reviewers. Any product that may be evaluated in this article, or claim that may be made by its manufacturer, is not guaranteed or endorsed by the publisher.
